# Supplementary material for: Bacterial meningitis in the early postnatal mouse studied at single-cell resolution
Source: eLife. 2023 Jun 15;12:e86130. doi: 10.7554/eLife.86130 (PMC10270687; doi:10.7554/eLife.86130)
Supplement: Supplementary file 1. [file elife-86130-supp1.doc]

**Supplemental Table 1.**

**Libraries**

number mean number of

library treatment of nuclei transcripts per nucleus

JW19 uninfected 7282 1680.02

JW20 uninfected 7074 1618.53

JW21 infected 8521 1476.26

JW22 infected 11195 1232.82

JW23 infected 14869 976.33

total 48941 1320

(1320 is the mean over all nuclei, not of the mean of the five samples.)

**Number of nuclei per cluster**

Cell type infected control total

Arachnoid barrier cells 1236 396 1632

Endothelial cells 3558 1154 4712

Fibroblasts, arachnoid 4043 2111 6154

Fibroblasts, dura 1 4280 1798 6078

Fibroblasts, dura 2 2767 1647 4414

Fibroblasts, dura 3 2857 1304 4161

Fibroblasts, mitotic 668 312 980

Fibroblasts, pia 5380 2199 7579

Glia 2103 377 2480

Immune cells 3790 1555 5345

Mural cells 437 242 679

Neurons 580 131 711

Osteoblasts 2010 923 2933

Osteoclasts 148 66 214

U1 468 119 587

U2 260 22 282

total 34585 14356 48941
