# Supplementary material for: Bacterial meningitis in the early postnatal mouse studied at single-cell resolution
Source: eLife. 2023 Jun 15;12:e86130. doi: 10.7554/eLife.86130 (PMC10270687; doi:10.7554/eLife.86130)
Supplement: Supplementary file 2. [file elife-86130-supp2.docx]

**Supplemental Table 2**

List of literature-based markers for characterization of cell identity of meningeal clusters

| Cell type | Gene | Ref. |
| --- | --- | --- |
| **Myeloid cells/Immune cells** | *Ptprc (Cd45)* | 1, 2 |
| Macrophages | *Csf1r* | 3 |
|  | *C1qa* | 3 |
|  | *F13a1* | 4 |
|  | *Adgre1* | 3, 5 |
|  | *Mrc1/Cd206* | 3, 6 |
|  | *Lyve1* | 3, 6 |
|  | *Apoe* | 3 |
|  | *Spi1* | 1 |
| CCL2+ macrophages | *Ccl2* | 2 |
| Monocytes/Monocytes-derived cells | *Ccr2* | 6, 7 |
|  | *H2-d1* | 8 |
|  | *Ly6c2* | 6, 9 |
|  | *S100a8* | 10 |
|  | *S100a9* | 10 |
|  | *Cytip* | 11, 12 |
| ILC2 | *Il7r* | 13 |
|  | *Gata3* | 13 |
|  | *Il1rl1* | 13 |
| ILC3 | *Rorc/Rorgt* | 13 |
| T cells | *Cd3e* | 2 |
|  | *Cd4* | 2 |
| Microglia | *Ccr5* | 14 |
|  | *Siglech* | 15 |
|  | *Hexb* | 16 |
|  | *P2ry12* | 17 |
| Osteoclasts | *Ctsk* | 18 |
|  | *Atp6v0d2* | 19, 20 |
|  | *Igtb3* | 21 |
| **Endothelial cells** | *Pecam1* | 22, 23 |
|  | *Erg* | 24 |
|  | *Tek* | 25 |
| Endothelial cell, arachnoid | *Cldn5* | 22, 26 |
|  | *Lef1* | 27, 28 |
|  | *Slc7a5* | 22 |
|  | *Lrp8* | 29 |
|  | *Slco1c1* | 30 |
| Endothelial cell, dura | *Plvap* | 31, 32 |
| Endothelial cell, dura, VWF+ | *Vwf* | 32, 33, 34 |
| Endothelial cell, artery | *Bmx* | 22, 35 |
|  | *Fbln5* | 23 |
|  | *Vegfc* | 22, 36 |
| **Mural cells** | *Abcc9* | 32 |
|  | *Notch3* | 37 |
|  | *Pdgfrb* | 32 |
| **Fibroblasts** | *Col1a1* | 38 |
|  | *Pdgfra* | 22, 38 |
| Fibroblast, pia | *Lama1* | 38 |
|  | *Cxcl12* | 38 |
|  | *Col15a1* | 39 |
| Fibroblast, arachnoid | *Nnat* | 38 |
|  | *Aldh1a2* | 38 |
|  | *Ptgds* | 38 |
| Fibroblast, dura | *Fxyd5* | 38 |
|  | *Foxp1* | 40 |
|  | *Mgp* | 38 |
| Arachnoid barrier | *Cdh1* | 38, 41 |
|  | *Cldh11* | 38 |
|  | *Tjp1* | 38 |
| **Osteoblasts** | *Runx2* | 42 |
|  | *Alpl* | 43 |
| **Neurons** | *Rbfox3* | 44 |
|  | *Nrxn3* | 45 |
| **Glia** | *Gfap* | 46 |
|  | *Aqp4* | 47 |
| **Mitotic cells** | *Top2a* | 48 |
|  | *Mki67* | 49 |

**Supplemental references**

1. Goldmann T, Wieghofer P, Jordão MJ, Prutek F, Hagemeyer N, Frenzel K, Amann L, Staszewski O, Kierdorf K, Krueger M, Locatelli G. Origin, fate and dynamics of macrophages at central nervous system interfaces. *Nat. Immunol.* **17**, 797-805 (2016).
2. Schafflick D, Wolbert J, Heming M, Thomas C, Hartlehnert M, Börsch AL, Ricci A, Martín-Salamanca S, Li X, Lu IN, Pawlak M. Single-cell profiling of CNS border compartment leukocytes reveals that B cells and their progenitors reside in non-diseased meninges. *Nat. Neurosci.* **24**, 1225-1234 (2021).
3. Van Hove H, Martens L, Scheyltjens I, De Vlaminck K, Pombo Antunes AR, De Prijck S, Vandamme N, De Schepper S, Van Isterdael G, Scott CL, Aerts J. A single-cell atlas of mouse brain macrophages reveals unique transcriptional identities shaped by ontogeny and tissue environment. *Nat. Neurosci.* **22**,1021-1035 (2019).
4. Kang B, Alvarado LJ, Kim T, Lehmann ML, Cho H, He J, Li P, Kim BH, Larochelle A, Kelsall BL. Commensal microbiota drive the functional diversification of colon macrophages. *Mucosal Immunol.* **13**, 216-229 (2020).
5. Schulz C, Perdiguero EG, Chorro L, Szabo-Rogers H, Cagnard N, Kierdorf K, Prinz M, Wu B, Jacobsen SE, Pollard JW, Frampton J. A lineage of myeloid cells independent of Myb and hematopoietic stem cells. *Science* **336**, 86-90 (2012).
6. Jordão MJ, Sankowski R, Brendecke SM, Sagar, Locatelli G, Tai YH, Tay TL, Schramm E, Armbruster S, Hagemeyer N, Groß O. Single-cell profiling identifies myeloid cell subsets with distinct fates during neuroinflammation. *Science* **363**, eaat7554 (2019).
7. Fantuzzi L, Borghi P, Ciolli V, Pavlakis G, Belardelli F, Gessani S. Loss of CCR2 expression and functional response to monocyte chemotactic protein (MCP-1) during the differentiation of human monocytes: role of secreted MCP-1 in the regulation of the chemotactic response. *Blood* **94**, 875-883 (1999).
8. Olingy CE, Dinh HQ, Hedrick CC. Monocyte heterogeneity and functions in cancer. *J. Leukoc. Biol.* **106**, 309–322 (2019).
9. Sunderkötter C, Nikolic T, Dillon MJ, Van Rooijen N, Stehling M, Drevets DA, Leenen PJ. Subpopulations of mouse blood monocytes differ in maturation stage and inflammatory response. *J. Immunol.* **172**, 4410–4417 (2004).
10. Averill MM, Kerkhoff C, Bornfeldt KE. S100A8 and S100A9 in cardiovascular biology and disease. *Arterioscler. Thromb. Vasc. Biol.* **32**, 223–229 (2012).
11. Boehm T, Hofer S, Winklehner P, Kellersch B, Geiger C, Trockenbacher A, Neyer S, Fiegl H, Ebner S, Ivarsson L, Schneider R. Attenuation of cell adhesion in lymphocytes is regulated by CYTIP, a protein which mediates signal complex sequestration. *EMBO J*. **22**, 1014-1024 (2003).
12. Hu C, Chu C, Liu L, Wang C, Jin S, Yang R, Rung S, Li J, Qu Y, Man Y. Dissecting the microenvironment around biosynthetic scaffolds in murine skin wound healing. *Sci. Adv.* **7**, eabf0787 (2021).
13. Yu Y, Tsang JC, Wang C, Clare S, Wang J, Chen X, Brandt C, Kane L, Campos LS, Lu L, Belz GT. Single-cell RNA-seq identifies a PD-1hi ILC progenitor and defines its development pathway. *Nature* **539**, 102-106 (2016).
14. He J, Chen Y, Farzan M, Choe H, Ohagen A, Gartner S, Busciglio J, Yang X, Hofmann W, Newman W, Mackay CR. CCR3 and CCR5 are co-receptors for HIV-1 infection of microglia. *Nature* **385**, 645-649 (1997).
15. Konishi H, Kobayashi M, Kunisawa T, Imai K, Sayo A, Malissen B, Crocker PR, Sato K, Kiyama H. Siglec‐H is a microglia‐specific marker that discriminates microglia from CNS‐associated macrophages and CNS‐infiltrating monocytes. *Glia* **65**, 1927-1943 (2017).
16. Masuda T, Amann L, Sankowski R, Staszewski O, Lenz M, Snaidero N, Costa Jordão MJ, Böttcher C, Kierdorf K, Jung S, Priller J. Novel Hexb-based tools for studying microglia in the CNS. *Nat. Immunol*. **21**, 802-815 (2020).
17. Lou N, Takano T, Pei Y, Xavier AL, Goldman SA, Nedergaard M. Purinergic receptor P2RY12-dependent microglial closure of the injured blood–brain barrier. *Proc Natl Acad Sci USA*  **113**, 1074-1079 (2016).
18. Lotinun S, Kiviranta R, Matsubara T, Alzate JA, Neff L, Lüth A, Koskivirta I, Kleuser B, Vacher J, Vuorio E, Horne WC. Osteoclast-specific cathepsin K deletion stimulates S1P-dependent bone formation. *J. Clin. Investig.* **123** (2013).
19. Lee SH, Rho J, Jeong D, Sul JY, Kim T, Kim N, Kang JS, Miyamoto T, Suda T, Lee SK, Pignolo RJ. v-ATPase V0 subunit d2–deficient mice exhibit impaired osteoclast fusion and increased bone formation. *Nat. Med*. **12**, 1403-1409 (2006).
20. Wu H, Xu G, Li YP. Atp6v0d2 is an essential component of the osteoclast‐specific proton pump that mediates extracellular acidification in bone resorption. *J. Bone Miner. Res.* **24**, 871-885 (2009).
21. Romeo SG, Alawi KM, Rodrigues J, Singh A, Kusumbe AP, Ramasamy SK. Endothelial proteolytic activity and interaction with non-resorbing osteoclasts mediate bone elongation. *Nat. Cell Biol.* **21**, 430-441 (2019).
22. Vanlandewijck M, He L, Mäe MA, Andrae J, Ando K, Del Gaudio F, Nahar K, Lebouvier T, Laviña B, Gouveia L, Sun Y. A molecular atlas of cell types and zonation in the brain vasculature. *Nature* **554**, 475-480 (2018).
23. Kalucka J, de Rooij LP, Goveia J, Rohlenova K, Dumas SJ, Meta E, Conchinha NV, Taverna F, Teuwen LA, Veys K, García-Caballero M. Single-cell transcriptome atlas of murine endothelial cells. *Cell* **180**, 764-779 (2020).
24. Nikolova-Krstevski V, Yuan L, Le Bras A, Vijayaraj P, Kondo M, Gebauer I, Bhasin M, Carman CV, Oettgen P. ERG is required for the differentiation of embryonic stem cells along the endothelial lineage. *BMC Dev. Biol.* **9**, 1-14 (2009).
25. Dumont DJ, Yamaguchi TP, Conlon RA, Rossant J, Breitman ML. tek, a novel tyrosine kinase gene located on mouse chromosome 4, is expressed in endothelial cells and their presumptive precursors. *Oncogene* **7**,1471-1480 (1992).
26. Nitta T, Hata M, Gotoh S, Seo Y, Sasaki H, Hashimoto N, Furuse M, Tsukita S. Size-selective loosening of the blood-brain barrier in claudin-5–deficient mice. *J. Cell Biol.* **161**, 653-660 (2003).
27. Behrens J, Von Kries JP, Kühl M, Bruhn L, Wedlich D, Grosschedl R, Birchmeier W. Functional interaction of β-catenin with the transcription factor LEF-1. *Nature* **382**, 638-642 (1996).
28. Hupe M, Li MX, Kneitz S, Davydova D, Yokota C, Kele J, Hot B, Stenman JM, Gessler M. Gene expression profiles of brain endothelial cells during embryonic development at bulk and single-cell levels. *Sci. Signal.* **10**, eaag2476 (2017).
29. Terstappen GC, Meyer AH, Bell RD, Zhang W. Strategies for delivering therapeutics across the blood–brain barrier. *Nat. Rev. Drug Discov*. **20**, 362-383 (2021).
30. Roberts LM, Woodford K, Zhou M, Black DS, Haggerty JE, Tate EH, Grindstaff KK, Mengesha W, Raman C, Zerangue N. Expression of the thyroid hormone transporters monocarboxylate transporter-8 (SLC16A2) and organic ion transporter-14 (SLCO1C1) at the blood-brain barrier. *Endocrinology* **149**, 6251-6261 (2008).
31. Stan RV, Kubitza M, Palade GE. PV-1 is a component of the fenestral and stomatal diaphragms in fenestrated endothelia. *Proc Natl Acad Sci USA* **96**, 13203-13207 (1999).
32. Rustenhoven J, Drieu A, Mamuladze T, de Lima KA, Dykstra T, Wall M, Papadopoulos Z, Kanamori M, Salvador AF, Baker W, Lemieux M. Functional characterization of the dural sinuses as a neuroimmune interface. *Cell* **184**, 1000-1016 (2021).
33. Rohlenova K, Goveia J, García-Caballero M, Subramanian A, Kalucka J, Treps L, Falkenberg KD, de Rooij LP, Zheng Y, Lin L, Sokol L. Single-cell RNA sequencing maps endothelial metabolic plasticity in pathological angiogenesis. *Cell metab.* **31**, 862-877 (2020).
34. Wang Y, Chen D, Xu D, Huang C, Xing R, He D, Xu H. Early developing B cells undergo negative selection by central nervous system-specific antigens in the meninges. *Immunity* **54**, 2784-2794 (2021).
35. Ekman N, Lymboussaki A, Västrik I, Sarvas K, Kaipainen A, Alitalo K. Bmx tyrosine kinase is specifically expressed in the endocardium and the endothelium of large arteries. *Circulation* **96**,1729-1732 (1997).
36. Hogan BM, Herpers R, Witte M, Heloterä H, Alitalo K, Duckers HJ, Schulte-Merker S. Vegfc/Flt4 signalling is suppressed by Dll4 in developing zebrafish intersegmental arteries. *Development* **136**, 4001-4009 (2009).
37. Liu H, Kennard S, Lilly B. NOTCH3 expression is induced in mural cells through an autoregulatory loop that requires endothelial-expressed JAGGED1. *Circ. Res.* **104**, 466-475 (2009).
38. DeSisto J, O’Rourke R, Jones HE, Pawlikowski B, Malek AD, Bonney S, Guimiot F, Jones KL, Siegenthaler JA. Single-cell transcriptomic analyses of the developing meninges reveal meningeal fibroblast diversity and function*. Dev. cell.* **54**, 43-59 (2020).
39. La Manno G, Siletti K, Furlan A, Gyllborg D, Vinsland E, Mossi Albiach A, Mattsson Langseth C, Khven I, Lederer AR, Dratva LM, Johnsson A. Molecular architecture of the developing mouse brain. *Nature* **596**, 92-96 (2021).
40. Dorrier CE, Jones HE, Pintarić L, Siegenthaler JA, Daneman R. Emerging roles for CNS fibroblasts in health, injury and disease. *Nat. Rev. Neurosci.* **23**, 23-34 (2022).
41. Derk J, Como CN, Jones HE, Joyce LR, Bonney S, O’Rourke R, Pawlikowski B, Doran KS, Siegenthaler JA. Formation and function of the meninges arachnoid barrier around the developing brain. *bioRxiv* (2022).
42. Ducy P, Zhang R, Geoffroy V, Ridall AL, Karsenty G. Osf2/Cbfa1: a transcriptional activator of osteoblast differentiation. *Cell* **89**, 747-754 (1997).
43. Golub EE, Boesze-Battaglia K. The role of alkaline phosphatase in mineralization. *Curr. Opin. Orthop*. **18**, 444-448 (2007).
44. Mullen RJ, Buck CR, Smith AM. NeuN, a neuronal specific nuclear protein in vertebrates. *Development* **116**, 201-211 (1992).
45. Chen YJ, Friedman BA, Ha C, Durinck S, Liu J, Rubenstein JL, Seshagiri S, Modrusan Z. Single-cell RNA sequencing identifies distinct mouse medial ganglionic eminence cell types. *Sci. Rep.* **7**, 1-1 (2017).
46. Eng LF. Glial fibrillary acidic protein (GFAP): the major protein of glial intermediate filaments in differentiated astrocytes. *J. Neuroimmunol*. **8**, 203-214 (1985).
47. Rash JE, Yasumura T, Hudson CS, Agre P, Nielsen S. Direct immunogold labeling of aquaporin-4 in square arrays of astrocyte and ependymocyte plasma membranes in rat brain and spinal cord. *Proc Natl Acad Sci USA***95**, 11981-11986 (1998).
48. De Resende MF, Vieira S, Chinen LT, Chiappelli F, da Fonseca FP, Guimarães GC, Soares FA, Neves I, Pagotty S, Pellionisz PA, Barkhordarian A. Prognostication of prostate cancer based on TOP2A protein and gene assessment: TOP2A in prostate cancer. *J. Transl. Med.* **11**, 1-9 (2013).
49. Gerdes J, Li L, Schlueter C, Duchrow M, Wohlenberg C, Gerlach C, Stahmer I, Kloth S, Brandt E, Flad H. Immunobiochemical and molecular biologic characterization of the cell proliferation-associated nuclear antigen that is defined by monoclonal antibody Ki-67. *Am. J. Pathol.* **138**, 867 (1991).
