# Supplementary material for: Bacterial meningitis in the early postnatal mouse studied at single-cell resolution
Source: eLife. 2023 Jun 15;12:e86130. doi: 10.7554/eLife.86130 (PMC10270687; doi:10.7554/eLife.86130)
Supplement: Supplementary file 4. [file elife-86130-supp4.doc]

**Supplemental Table 4.**

**Figure Number of mice quantified**

**Control Infected**

2E (PU.1) 3 3

2I (Leptomeninges, LYVE1) 3 3

2I (Leptomeninges,CD206) 3 3

2I (Dura, LYVE1) 4 3

2I (Dura, CD206) 4 3

2I (Leptomeninges, CD180) 3 3

2I (Dura, IL-6) 4 3

2K (Dura, S100A8) 3 3

2K (Leptomeninges, S100A8) 2 2

5C (CLDN5) 3 3

5C (PECAM1) 3 3

5C (CLDN5, LPS) 3 3

5C (Sulfo-NHS-biotin) 5 5

5D (CLDN5 Western blot) 4 4

5G (LEF1 Western blot) 3 3

5H (VEGFR2) 3 3

5H (LEF1) 3 3

5H (ERG) 3 3

6B (angle of EC nuclei) 3 3

6F (Dura) 2 2

6F (Leptomeninges) 2 2

7B (area covered, each group) 3 3

7B (PU.1, each group) 3 3

7B (Sulfo-NHS-biotin, C57) 3 3

7B (Sulfo-NHS-biotin, *Tlr4*-/-) 4 4

7B (Sulfo-NHS-biotin, Ccr2-/-) 2 3

8D (area, each group) 3 3

8D (HA+/ERG+, each group) 2 2

8D (S-NHS-biotin, each group) 4 3

**Clodronate experiment (Figure 8D)**

8C (CD206, Dural sinus) Control=2 ICV=3 CM=3

8C (CD206, Dura no sinus) Control=3 ICV=3 CM=3

8C (CD206, Leptomeninges) Control=4 ICV=4 CM=4

**Supplemental Figure 3**

3A (weight) Control=17 Infected=16

3B (survival curve) Control=11 Infected=11

3C (bacterial number, FVB/NJ) Dura= 11 Leptomeninges=11

3C (C57, *Tlr4*-/-, *Ccr2*-/-) C57=10 *Tlr4*-/-=10 *Ccr2*-/-=10

3C (Liposomes) Control liposomes= 6 Clodronate liposome=8
